# Graphene Quantum Dots as Nanozymes for Electrochemical Sensing of *Yersinia enterocolitica* in Milk and Human Serum

**DOI:** 10.3390/ma12132189

**Published:** 2019-07-08

**Authors:** Sumeyra Savas, Zeynep Altintas

**Affiliations:** 1 National Research Institute of Electronics and Cryptology, The Scientific and Technological Research Council of Turkey (TUBITAK), Kocaeli 41470, Turkey; 2 Institute of Chemistry, Technical University of Berlin, Straße des 17. Juni 124, Berlin 10623, Germany

**Keywords:** *Yersinia enterocolitica*, pathogen detection, graphene quantum dots (GQDs), GQD-immunosensor, enzyme mimics, infectious diseases

## Abstract

The genus *Yersinia* contains three well-recognized human pathogens, including *Y. enterocolitica*, *Y. pestis*, and *Y. pseudotuberculosis*. Various domesticated and wild animals carry *Yersinia* in their intestines. Spread to individuals arises from eating food or water contaminated by infected human or animal faeces. Interaction with infected pets and domestic stock may also lead to infection. *Yersinia* is able to multiply at temperatures found in normal refrigerators; hence, a large number of the bacteria may be present if meat is kept without freezing. *Yersinia* is also rarely transmitted by blood transfusion, because it is able to multiply in stored blood products. Infection with *Yersinia* can cause yersiniosis, a serious bacterial infection associated with fever, abdominal pain and cramps, diarrhea, joint pain, and symptoms similar to appendicitis in older children and adults. This paper describes a novel immunosensor approach using graphene quantum dots (GQDs) as enzyme mimics in an electrochemical sensor set up to provide an efficient diagnostic method for *Y. enterecolitica*. The optimum assay conditions were initially determined and the developed immunosensor was subsequently used for the detection of the bacterium in milk and human serum. The GQD-immunosensor enabled the quantification of *Y. enterocolitica* in a wide concentration range with a high sensitivity (LOD_milk_ = 5 cfu mL^−1^ and LOD_serum_ = 30 cfu mL^−1^) and specificity. The developed method can be used for any pathogenic bacteria detection for clinical and food samples without pre-sample treatment. Offering a very rapid, specific and sensitive detection with a label-free system, the GQD-based immunosensor can be coupled with many electrochemical biosensors.

## 1. Introduction

*Yersinia enterocolitica* is a gram-negative bacillus shaped bacterium that leads to a zootonic disease called yersiniosis. The infection is demonstrated as mesenteric adenitis, acute diarrhea, terminal ileitis, and pseudoappendicitis. Rarely, it can even result in sepsis [1]. According to the 2017 report of the European Food Safety Authority (EFSA) and European Centre for Disease Prevention and Control (ECDC), *Y. enterocolitica* has been realized as the third most common foodborne-zoonotic disease after campylobacteriosis and salmonellosis in the European Union [2]. 

Nutrients are stored in refrigerators for a long time at 4 °C and *Y. enterocolitica* has the ability to quickly and easily grow and proliferate at this temperature. This situation increases the risk of infection via contaminated food and dairy products [1]. The bacterium is transmitted to humans by contaminated water and food. Raw and pasteurized milk, as well as various vegetables, may be particular sources of this pathogen [1]. In 1982, *Y. enterocolitica* was found in pasteurized milk and led to an outbreak in Arkansas [3]. Several studies suggested that the bacterium cannot survive after a proper pasteurization process [4,5], although contrary findings were also reported [6]. The quick and accurate detection of the bacterium from food products or the body fluids of infected individuals is, therefore, important [1].

Several techniques have been used for the detection of *Y. enterocolitica*, including cultivation methods [7], serological tests [8], enzyme link immunosorbent assay (ELISA) [9], and polymerase chain reaction (PCR)-based tests [10,11]. Despite being well-established techniques, they are subject to some major limitations associated with assay complexity, the necessity for a long analysis time, and the possibility of obtaining false positive results due to the cross-reactions with other infectious diseases. Biosensors offer strong alternatives to these techniques for rapid and sensitive quantification of *Y. enterocolitica*. Various transducer systems, such as surface plasmon resonance (SPR) [12,13], fiber optic [14], and electrochemical [15,16] sensors, have been used for this aim. Although the applications of sensor technologies in pathogenic bacteria determination are very common, the number of *Yersinia* sensors reported in the literature are limited to a few examples. More importantly, these sensors were not coupled with the nanomaterials that provide significantly higher sensitivity, specificity, and reproducibility than those of traditional sensors. 

Among the nanomaterials, graphene quantum dots (GQDs) are particularly attractive for label-free detection techniques for microorganisms [17,18]. They are defined as zero-dimensional (0D) materials with characteristics derived from both graphene and carbon dots, which can be considered exceedingly small pieces of graphene. By turning 2D graphene sheets into 0D GQDs, the GQDs display new phenomena owing to quantum confinement and edge effects, which are akin to carbon dots [19]. Compared with semiconductive quantum dots and organic dyes, GQDs are superior in terms of their exceptional properties, such as biocompatibility, low toxicity, and high photostability against photobleaching and blinking [18,19]. Unlike carbon dots, GQDs clearly possess a graphene structure inside the dots, irrespective of the dot size, which confers them with some of the unusual properties of graphene [19]. GQDs have, therefore, attracted significant attention from researchers in different fields since 2000 [17,18,19,20,21].

GQDs possess peroxidase (POD)-like catalytic activities, where the reactions involve the oxidation of an electron-donor substrate with the concurrent reduction of hydrogen peroxide (H_2_O_2_). [19,22,23] Horseradish peroxidase (HRP) is the most commonly used peroxidase in biosensing applications, which require the labelling of a secondary receptor for target determination, thus making the assay procedures time-consuming and costly. Acting as nanozymes, HRP-based systems can be replaced by GQDs that allow the label-free detection of analytes. Guo et al. assembled the GQDs on a gold electrode, where the GQDs showed highly peroxidase-like properties even after being assembled on gold. The as-prepared GQDs-Au electrode showed good electrochemical catalytic properties toward the H_2_O_2_ decomposition. Electrochemical measurements showed that the GQDs-Au electrode bears a fast amperometric response to H_2_O_2_, a wide linear range, and a low detection limit [22]. Yang et al. recently reported a label-free electrochemical immunosensor, where the GQDs efficiently reduced H_2_O_2_ and allowed the sensitive detection of carcinoembryonic antigen (CEA) in human serum [23].

**Scheme 1:**
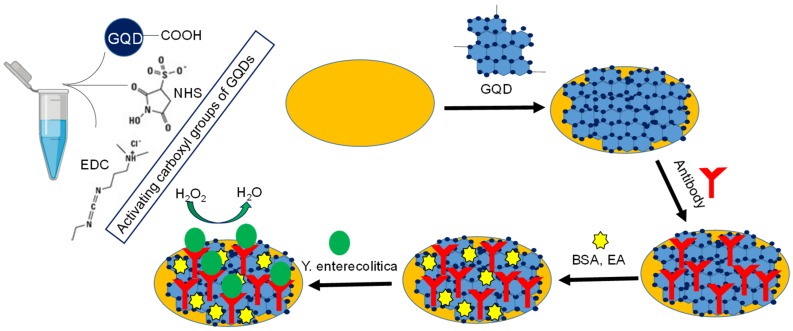
Principle of the graphene quantum dots (GQDs)-based immunosensor for *Y. enterocolitica* detection.

GQDs have the ability to increase the electrochemical reaction speed and this is particularly important to consider while developing electrochemical sensors [19]. To avoid sample pre-treatment and bestow a rapid and easy-to-apply detection principle, we have developed an immunoassay with GQDs due to their aforementioned characteristics. We introduced an electrochemical sensing approach for highly sensitive and specific determination of *Y. enterocolitica* in milk and human serum for the first time. To this end, the activated GQDs were laminated on a gold electrode prior to the antibody immobilization, followed by inactivating unreacted carboxyl groups on the surface with bovine serum albumin (BSA) and ethanolamine (EA) (Scheme 1). The quantification of bacterium was achieved based on the reduction of H_2_O_2_ by GQDs. The bare gold electrode surface could not reduce H_2_O_2_ itself, whereas the GQD-modified gold electrode significantly reduced H_2_O_2_ and generated gradually increasing signals at mA range based on the increasing GQD concentration used. The enhanced electrocatalytic property of the GQD-modified electrode could be attributed to the intimate electronic interactions between Au and GQDs, which improves electron transfer. The detection of bacteria was based on the degree of inhibited electron transfer of the laminated GQDs on the Au surface, which was hampered by the formation of antigen–antibody complex on the GQD-modified Au electrode. Thus, the signal response of the immunosensor decreased along with the increased *Y. enterecolitica* concentration in the sample solution. To best of our knowledge, we could achieve the highest sensitivity and specificity for *Y. enterocolitica* by using the label-free, real time GQDs-based electrochemical sensor. This may help in the identification of possible infectious diseases and any pathogenic bacteria which exist in food products. 

## 2. Materials and Methods

### 2.1. Materials and Reagents

A monoclonal anti-*Y. enterocolitica* antibody was bought from BIO-RAD (Puchheim, Germany). Heat-killed *Y. enterocolitica*, *Salmonella typhimurium*, *Bacillus anthracis*, *Y. pestis* and *Eschericia coli* were purchased from SeraCare Life Sciences (Gaithersburg, MD, USA). Sterile human serum, 1-Ethyl-3-(3-dimethylaminopropyl)-carbodiimide (EDC), N- hidroxysuccinimide (NHS), ethanolamine, analytical grade ethanol, hydrogen peroxide, phosphate-buffered saline tablets (PBS, 0.01 M), graphene quantum dots (<5 nm) were bought from Sigma Aldrich (Poole, UK). Milk was obtained from a local market. Bovine serum albumin (BSA) was purchased from Merck (Darmstadt, Germany). Double distilled water was produced by a Milli-Q water system (Millipore Corp., Tokyo, Japan).

### 2.2. Fabrication, Cleaning and Coating of Working Electrodes

**Figure 1:**
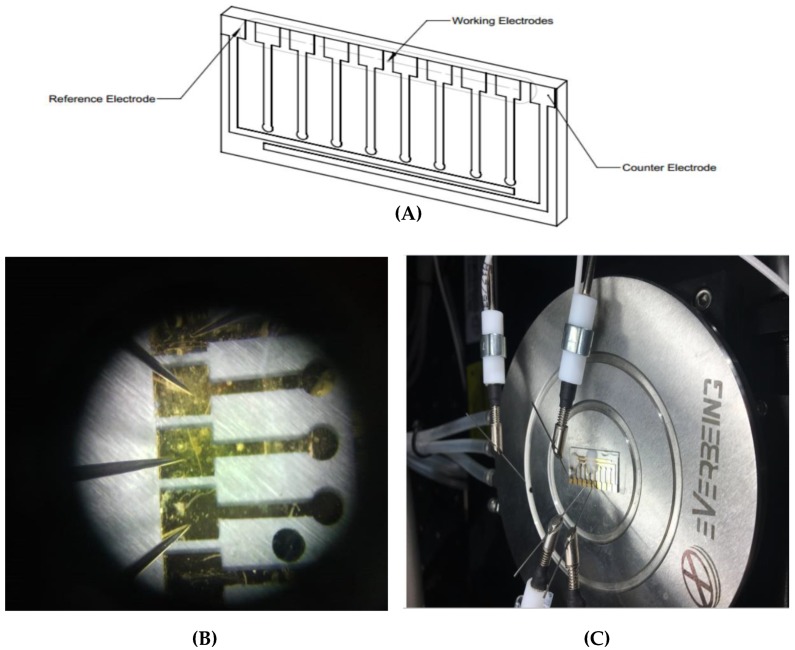
(**A**) Illustration of electrodes. (**B**) A picture of electrodes. (**C**) Electrochemical measurement set up of Keithley-4200 semi-conductor parameter analyzer KTE I version (V9.1).

The electrodes were laid out on the glass slide using a fine metal mask, which was made of a laser-cut patterned stainless steel. Gold was deposited on the wafer using an electron beam evaporator (Ebeam system Nanovak NVEB-600, Nanovak, Ankara, Turkey). Prior to the application of Au (200 nm), a 40 nm Ti layer was implemented onto the wafer as an intermediary adhesive layer to enhance the adhesion between the glass slide and the Au [24]. The electrodes were then washed with double distilled water and dried thoroughly under a stream of nitrogen gas. Electrochemical measurements were performed using a Keithley-4200 semi-conductor parameter analyzer KTE I version (V9.1). The illustration of electrodes and the measurement set up are presented in Figure 1.

The electrodes were cleaned by employing nitrogen plasma prior to laminating the EDC-NHS activated GQDs on the sensing surfaces [25]. The GQDs, diluted in double distilled water, were mixed with EDC (0.4 M)-NHS (0.1 M) solution in a tube, which was stirred on a shaker for 8 h at 4 °C. A 200 µL of this solution was then injected onto the gold working electrodes and incubated overnight to laminate the GQDs on the surface. 

### 2.3. Determination of Optimal GQDs Concentration for Bioassays

The morphology and uniformity of GQDs were initially characterized using a transmission electron microscope (TEM, JEM-2100, JEOL Ltd. Tokyo, Japan). The GQD sample was prepared by filtering 10 μL through a 1.2 μm glass fiber syringe filter and depositing the filtrate on a silicon chip attached to a TEM holder, and leaving them to dry overnight in a fume hood prior to measurement. Six different concentrations of GQDs (0.5, 5, 50, 500, 5000 and 50,000 ppm) were prepared in double distilled water and then measured on the bare electrode surface to determine the optimum GQD concentration based on the signal/concentration relationship towards the reduction of 5 mM H_2_O_2_. The experiments for each concentration were repeated three times and the optimum GQD concentration was determined for bioassays according to average electrochemical sensor signals. 

### 2.4. Antibody Immobilization and Characterization

The GQD-laminated sensor surface was washed with PBS buffer (pH: 7.4) prior to the covalent immobilization of the anti-*Y. enterocolitica* antibody (50 μg mL^−1^, prepared in NaAc buffer, pH 4.5) during 1 h incubation. To inactivate the remaining carboxyl groups on the surface and avoid possible non-specific interactions during the analyte detection assays, the electrodes were incubated with BSA (100 μg mL^−1^, prepared in PBS buffer) and ethanolamine (1 M, prepared in double distilled water, pH 8.5) for 4 min each. Each step of antibody immobilization was performed at room temperature in a Petri dish to protect it from dust or evaporation. All reagents (antibody, BSA, ethanolamine) were applied to the sensor surface at 200 µL volume. Between the subsequent steps the sensor surface was carefully rinsed with PBS buffer (pH 7.4). The bare gold, GQD-laminated, and antibody-immobilized sensor surfaces were characterized by an atomic force microscope (AFM) (Naio, Nanosurf AG, Liestal, Switzerland). Commercially available AFM probes (NCLR) from NanoWorld (NanoWorld AG, Liestal, Switzerland) were used for the measurements at a spring constant of 23.191 N/m, resonant frequency of 170,481 Hz, scan size of 3.027 µm, and scan speed of 2 s. All AFM measurements were carried out at room temperature using the intermittent air mode. 

### 2.5. Development of the Bioassay for Milk Samples

The initial assays were carried out in PBS buffer in a concentration range of 1–6.23 × 10^8^ cfu mL^−1^ using 5000 ppm of GQDs. For the assays in milk (50%, diluted with buffer), two sets of samples were prepared to determine the most convenient GQD concentration that is necessary for the quantification of *Y. enterocolitica* at lowest possible level. One set of milk samples contained 6 cfu mL^−1^ of the bacterium, whereas the second set comprised of 6.23 × 10^8^ cfu mL^−1^. Each sample set was measured on two different electrode surfaces, which were prepared with GQDs at a concentration of 5000 or 50,000 ppm. The bacteria samples (200 µL) were incubated on the anti-*Y. enterocolitica* antibody immobilized electrode surfaces for 8 min, washed with PBS prior to the amperometric measurements at −0.2 V in the presence of H_2_O_2_ (5 mM in PBS) [23]. Accordingly, the detection of bacterium in a wide concentration range was investigated using the electrodes prepared with 5000 ppm GQDs.

### 2.6. Development of the Bioassay for Human Serum Samples

The optimum concentration of GQDs for serum assays was investigated as described in Section 2.5. As the lower amount of GQD (5000 ppm) did not allow the quantification of the bacterium at 6 cfu mL^−1^, the higher GQD concentration (50,000 ppm) was selected. The quantification of *Y. enterocolitica* from 1 to 6.23 × 10^8^ cfu mL^−1^ was then studied using the GQD immunosensor. Each sample was injected onto the antibody immobilized surface at a volume of 200 µL, incubated for 8 min, and measured electrochemically in the presence of H_2_O_2_ (5 mM in PBS). All bioassays were performed using 50% of human serum diluted with buffer.

## 3. Results and Discussions

### 3.1. Determination of Optimal GQDs Concentration for Bioassays

**Figure 2:**
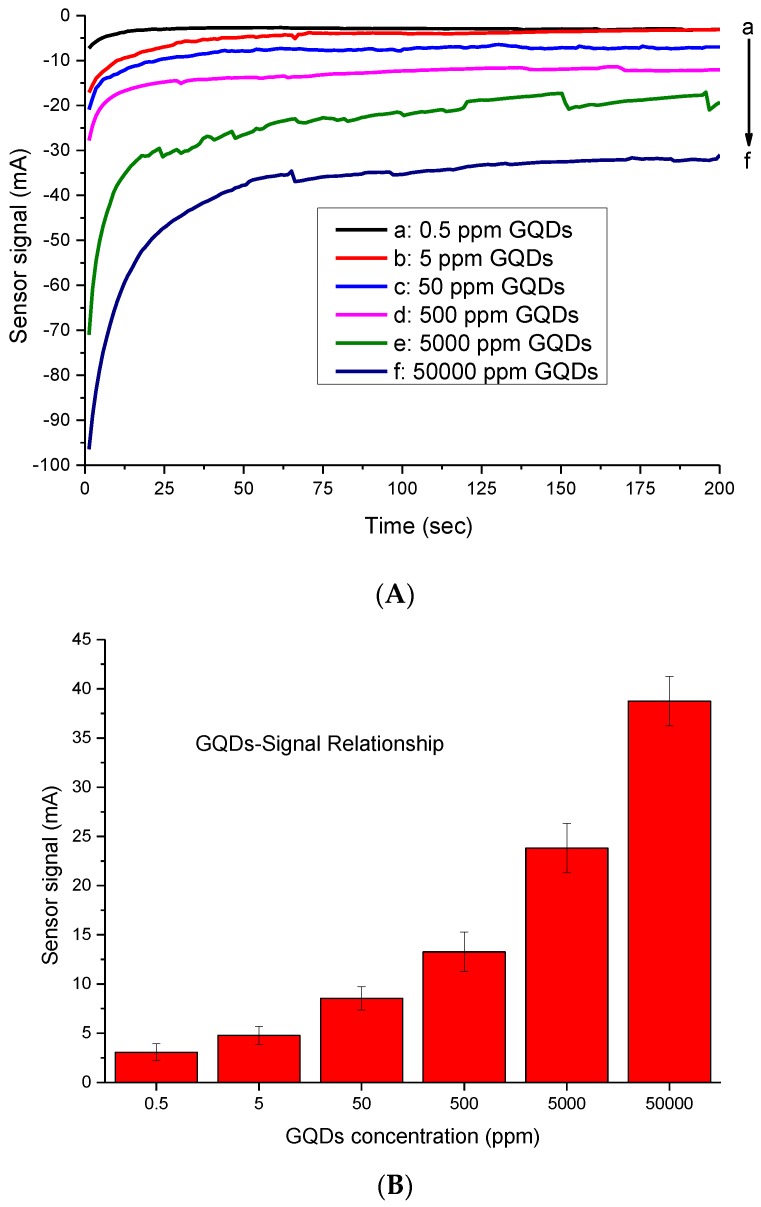
**(A)** Real-time measurement curves obtained with six different GQD concentrations. (**B**) Overall results of GQD optimization assays in a concentration range of 0.5–50,000 ppm (*n* = 3).

In this study, GQDs (<5 nm) were used as nanozymes replacing enzymatic systems. The intimate electronic interactions between the Au electrode and GQDs supplied high electrical conductivity and catalytic surface area, which turned them into the exceedingly active electrocatalysts for H_2_O_2_ reduction [22,23]. In this study, we first determined the convenient amount of GQDs for use in sensor development, based on the signal–concentration relationship. The GQDs resulted in sensor signals at mA range on the bare gold electrodes surfaces. Among the six different concentrations tested, 5000 ppm was initially selected for use in bioassays. This concentration led to 23.8 ± 2.5 mA response, which is significantly higher than normal bioassay systems, where the measured concentration of an analyte generally results in a response ranging from µA to nA. Although the higher GQDs concentration (50,000 ppm) led to a 38.76 ± 2.6 mA response, it was not chosen because this would have increased the cost of the developed system (Figure 2). 

**Figure 3:**
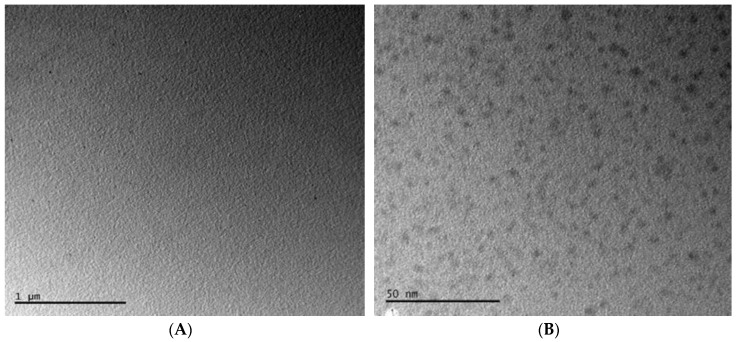
Characterization of GQDs using TEM at 1 µm (**A**) and 50 nm (**B**) scales.

The uniform size and morphology of the GQDs were confirmed by employing TEM (Figure 3).

### 3.2. Label Free Direct Assay for Y. enterocolitica Determination in Milk and Human Blood

**Figure 4:**
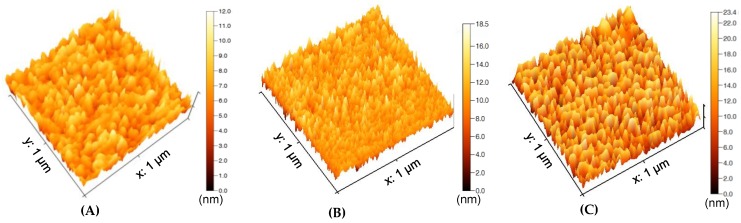
AFM analysis of bare (**A**), GQD-laminated (**B**), and antibody-immobilized (**C**) sensor surfaces.

The initial investigations for *Y. enterocolitica* detection were carried out in buffer to establish the bioassays. The bacterium specific antibody was covalently immobilized to the GQD-laminated Au electrode surface. The bare, GQD-laminated, and antibody-immobilized sensor surfaces were characterized by AFM (Figure 4). The 3D surface topology images in 1 × 1 µm^2^ scanning area resulted in the heights of 12 nm, 18.5 nm, and 23.4 nm for bare (Figure 4A), GQD-laminated (Figure 4B), and antibody-immobilized (Figure 4C) surfaces, respectively. The gradual increase of the surface height confirmed the successful preparation of the sensing surface for bacteria detection assays [26]. 

**Figure 5:**
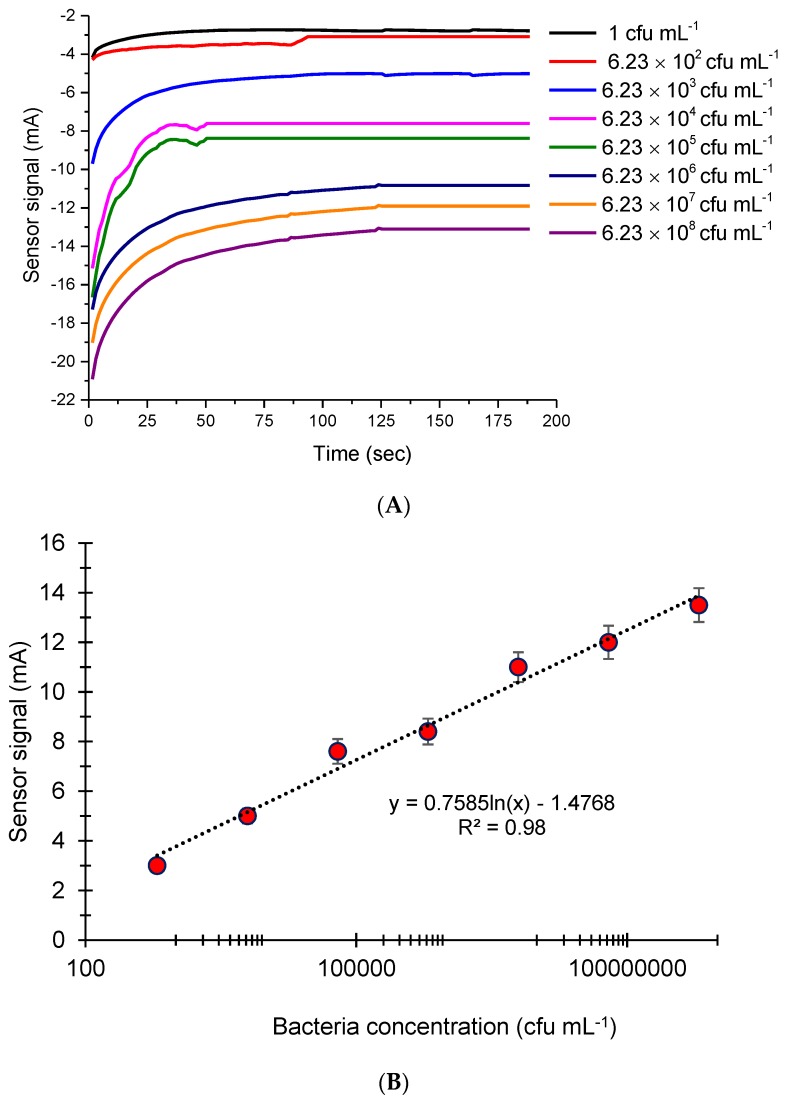
(**A**) The real-time measurement curves obtained for different concentrations of *Y. enterocolitica* in PBS buffer, using 5000 ppm GQDs as enzyme mimics. (**B**) Overall results of *Y. enterocolitica* detection in a concentration range of 6.23 × 10^2^–6.23 × 10^8^ cfu mL^−1^ with an R^2^ value of 0.98 (*n* = 3).

Eight *Y. enterocolitica* samples (1–6.23 × 10^8^ cfu mL^−1^) were prepared in PBS buffer (pH 7.4). These samples were then injected onto the sensor surface and the measurements were carried out using a three electrode system connected to a Keithley-4200 semi-conductor parameter analyzer. A 5000 ppm concentration of GQDs was sufficient to quantify 1 cfu mL^−1^ of *Y. enterocolitica* in 200 µL (the actual LOD was 5 cfu mL^−1^). However, the lower GQDs concentration (i.e., 500 ppm) could not provide this sensitivity level. The individual real time measurement results of bacteria detection are given in Figure 5A. The overall results of the assays in PBS buffer were subjected to logarithmic regression analysis, revealing R^2^ values of 0.98 and 0.92 in the concentration range of 6.23 × 10^2^ ‒6.23 × 10^8^ cfu mL^−1^ and 1–6.23×10^8^ cfu mL^−1^, respectively (Figure 5B). The lowest concentration of the bacterium generated a 2.8 ± 0.1 mA signal in buffer, which is significantly higher than those of earlier studies (~20 nA for 50 cfu mL^−1^ of *E. coli*), where HRP was used as the enzyme label in AuNP amplified sandwich assay in buffer [27]. This confirms the excellent properties of GQDs as enzyme mimics for biosensing applications. Moreover, the current bioassay strategy does not require the conjugation of nanomaterials with a secondary antibody, which decreases the cost and provides easy-to-apply sensor arrays [24,27]. 

**Figure 6:**
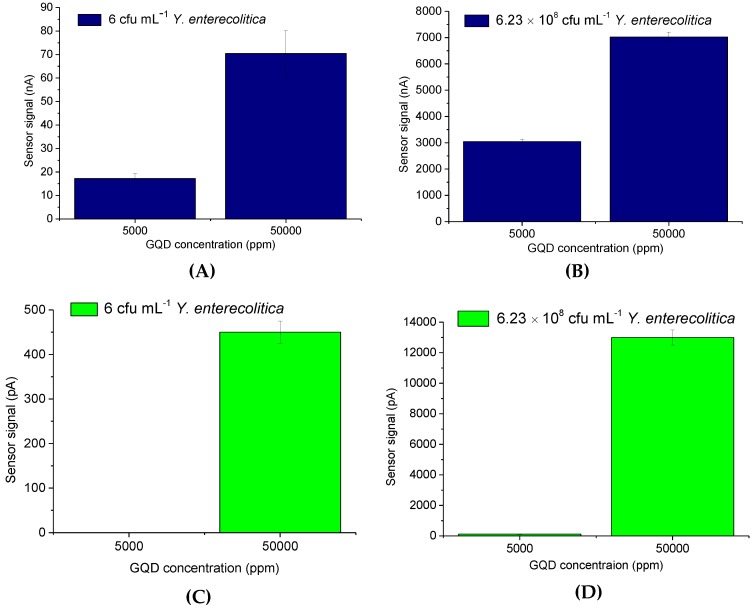
Optimization of GQD concentration for milk (**A**, **B**) and serum (**C**, **D**) assays by testing two different concentrations (6 and 6.23 × 10^8^ cfu mL^−1^) of *Y. enterecolitica* (*n* = 3).

To ensure the optimum GQD amount for milk and serum assays, we first tested two different concentrations of *Y. enterecolitica* (6 and 6.23 × 10^8^ cfu mL^−1^) by using the working electrodes prepared with 5000 or 50,000 ppm GQD. The lower GQD concentration (5000 ppm) was selected for milk assays, as it allowed the quantification of 6 cfu mL^−1^ of the bacterium with a sufficient sensor signal (17.2 ± 2.1 nA). On the other hand, this GQD concentration did not generate a signal in human serum for 6 cfu mL^−1^ of *Y. enterecolitica* and led to only 120 ± 20 pA signal for the highest concentration of the bacterium (6.23 × 10^8^ cfu mL^−1^). This is attributed to the presence of various interfering compounds in human serum, such as hormones, minerals, and proteins. Based on the preliminary tests, the assays in milk and serum were performed using the GQD concentrations of 5000 and 50,000 ppm, respectively (Figure 6). 

**Figure 7:**
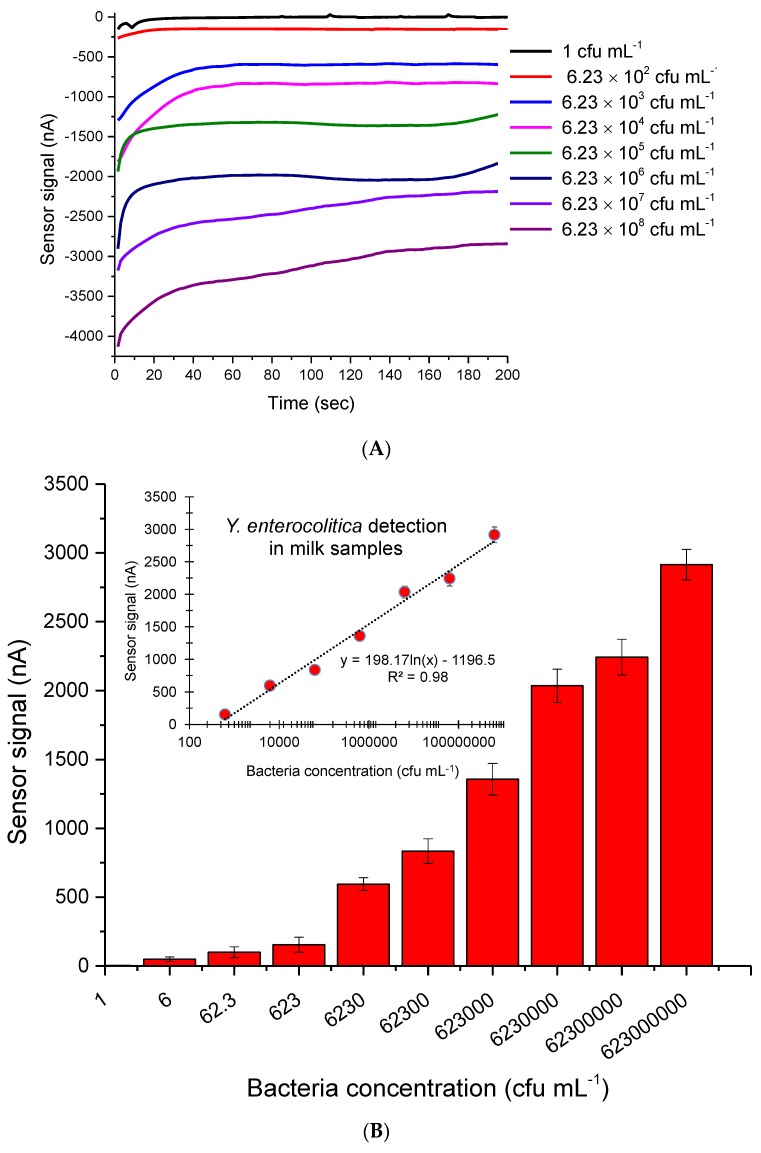

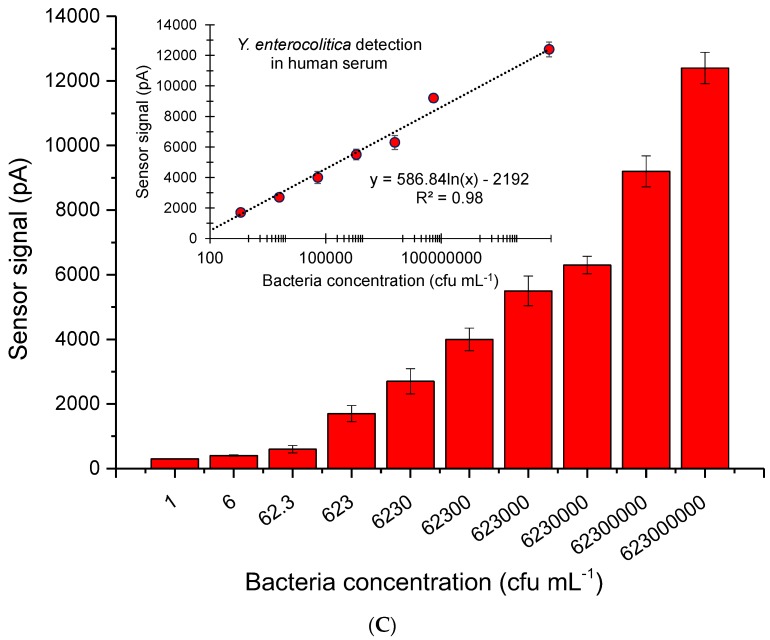
(**A**) The real-time measurement curves obtained for different concentrations of *Y. enterocolitica* in milk, using 5000 ppm GQDs as enzyme mimics. (**B**) The quantification of *Y. enterocolitica* in milk; inset: Regression analysis resulting in R^2^ value 0.98 (*n* = 6). (**C**) Overall results of *Y. enterocolitica* detection in human serum; inset: Regression analysis resulting in R^2^ value of 0.98 (*n* = 6). The bacterium was measured in a concentration range of 1‒6.23×10^8^ cfu mL^−1^ for both assays.

Ten different concentrations of the bacterium, from 1 to 6.23 × 10^8^ cfu·mL^−1^, were spiked in milk and serum. Each sample was injected to the antibody immobilized sensor for amperometric measurements at −0.2V. The lowest amounts of *Y. enterocolitica* measured in milk and serum were 1 and 6 cfu mL^−1^, respectively. The sensor generated 0.6 and 7.73 nA signals for only milk and 1 cfu mL^−1^ bacterium in milk, respectively. The negative control (serum) and 6 cfu mL^−1^ of *Y. enterocolitica* in serum resulted in 0.22 and 0.329 nA sensor signals, respectively. It is worth noting that all samples were prepared as 1 mL solutions and only 200 µL of the samples were injected to the electrode surface for measurements. Therefore, the actual LODs of milk and serum assays were determined to be 5 and 30 cfu mL^−1^, respectively. The overall results of bioassays for milk and serum samples are given in Figure 7. The correlation coefficient of both assays was found to be 0.98 when the data were subjected to the logarithmic regression analysis (Figure 7). 

The current methods for *Y. enterecolitica* detection include cultivation [7], ELISA [9], PCR [10,11], and SPR-based tests [12,13]. These techniques are either labor intensive or time consuming, and lack the desired sensitivity. The limit of detection of these methods varies between 10^2^‒10^6^ cfu mL^−1^ (Table 1). A few electrochemical sensors have been reported for the detection of *Yersinia* species, targeting bacteria specific genes [15,16,28]. The direct detection of bacteria using electrochemical transducers has not been studied so far. Additionally, although the nanomaterials integrated sensors have dominated in pathogenic bacteria detection in recent years [22,25,29,30], to best of our knowledge, such a system has not been introduced for *Yersinia* determination. Compared to our earlier works [24,27], reporting on the quantification of *E. coli* in water (LOD: 50 cfu·mL^−1^) and *Salmonella typhimirium* in human stool (LOD: 12 cfu mL^−1^) using gold nanoparticles amplified electrochemical sensors, we achieved higher sensitivities (LOD_milk_= 5 cfu mL^−1^, LOD_serum_= 30 cfu mL^−1^) in the current work, despite working with more complex media. It is worth noting that we performed a static assay procedure in the current work with a limited sample volume. In case of applying the developed strategy for fully automated microfluidic systems, we expect the sensitivity of the assays to increase, which may lead to quantifying one single bacterium. The current work allows label-free detection of *Y. enterocolitica* with a direct assay methodology, not requiring enzyme labels and detector antibody. The superior features of GQD-immunosensor supply a much shorter assay time than those of previous studies [24,27]. 

### 3.3. Cross-Reactivity Studies for Y. enterocolitica

To determine the specificity of the developed assay for *Y. enterocolitica*, the interaction of non-specific bacteria with the bacterium-specific antibody-immobilized sensor was studied. For this, the reference samples of *S. enteritidis, B. anthracis, E. coli*, and *Y. peptis* were separately prepared in milk and human serum at a fix concentration of 10^7^ cfu mL^−1^. Each sample was injected to the antibody immobilized surfaces and measured during 200 s. An extremely low cross-reactivity was recorded for all reference pathogens in milk, suggesting a very high specificity of the bioassay for the target bacterium (Table 2). The relative cross-reactivities of the non-specific pathogenic bacteria were found to be higher in human serum (Table 3) compared to milk. However, the maximum cross-reaction was still at an acceptable level (22.8%), which was recorded for another *Yersinia* species. It is also worth noting that the selected concentration of bacteria in cross-reactivity tests was quite high. 

To realize the applicability of the GQD-immunosensor in the co-existence of various bacteria species, we mixed the target bacterium and non-specific bacteria at equal concentrations (10^7^ cfu·mL^−1^), and subsequently measured the sensor signal to determine the relative activity for *Y. enterecolitica*. Considering that *Y. pestis* led to higher cross-reactivity for individual tests as being another *Yersinia* genus, we prepared two different mixtures of non-specific bacteria: 1) A mixture of *S. enteritidis*, *E. coli*, and *B. anthracis*; and 2) a mixture of *S. enteritidis*, *E. coli*, *B. anthracis*, and *Y. pestis*. The relative activity of the sensor for milk assays decreased only 2.2% and 5.5% in mixtures 1 and 2, respectively (Table 2). The suppression of the sensor signal in serum was determined to be higher, with 12.5% and 24.2% decrease in relative activity in mixtures 1 and 2, respectively (Table 3). Nevertheless, these results suggest that GQD-immunosensor is capable of quantifying the target bacterium at high specificity even in the co-existence of several non-specific bacteria. 

## 4. Conclusions

In this work, a novel GQD-based immunosensor was developed using an electrochemical transducer. Among all methods reported for the detection of *Y. enterecolitica*, the GQDs-enriched sensor supplied the highest selectivity and sensitivity in complex media, such as milk (LOD: 5 cfu·mL^−1^) and human serum (LOD: 30 cfu·mL^−1^). The developed method can be used for any pathogenic bacteria detection for clinical and food samples without pre-sample treatment. Offering a very rapid, specific and sensitive detection with a label-free system, the GQD-based immunosensor can be coupled with various electrochemical biosensors.

## Figures and Tables

**Table 1:** Comparison of various techniques for the detection of *Y. enterecolitica*.

Method	Sample	Ligand	Investigation Range	Detection Limit	Ref
ELISA	Dairy product	Antibody	5‒1000 cfu g^−1^	10 cfu g^−1^	[9]
Chemiluminescent ELISA	Human fecal/ bovine meat	Antibody	10^2^‒10^8^ cfu mL^−1^	10^4^‒10^5^ cfu mL^−1^	[31]
ELISA	Pig diaphragm muscle samples	Antibody	2‒4.75 log_10_ cfu g^−1^	3.56 log_10_ cfu g^−1^	[32]
Multiplex PCR	Pig feces	*Ail* gene	P/N	P/N	[33]
Real-time PCR and ELISA	Wild boar tonsils/feaces	*Ail* gene	P/N	P/N	[34]
SPR sensor	Buffer	Protein G	10^2^‒10^7^ cfu mL^−1^	10^2^ cfu mL^−1^	[12]
Imaging ellipsometry immunosensor	Buffer	Antibody	10^3^‒10^7^ cfu mL^−1^	10^3^ cfu mL^−1^	[13]
DNA microarray chip hybridization	Various food matrices	DNA	10^3^‒10^6^ cfu g^−1^	10^4^ cfu g^-1^ *	[35]
GQD sensor	Milk	Antibody	1‒6.23 × 10^8^ cfu mL^−1^	5 cfu mL^−1^	CW
GQD sensor	Human serum	Antibody	1‒6.23 × 10^8^ cfu mL^−1^	30 cfu mL^−1^	CW

P/N—positive/negative; *, the *ail* gene of bacterium was quantified; CW—current work.

**Table 2:** Current response profile for *Y. enterocolitica* binding in milk compared with other pathogenic bacteria, and the percentage of cross reactivity of non-specific bacteria (*n* = 3).

Bacteria Tested (10^7^ cfu mL^−1^)	Response (Current, nA)	GQD Immunosensor % Relative Activity
Yersinia enterocolitica	1473.0	100.0 ± 2.0
Yersinia pestis	93.2	6.0 ± 2.0
Salmonella enteritidis	27.4	1.9 ± 0.2
Escherichia coli	9.2	0.6 ± 0.1
Bacillus anthracis	1.0	0.0 ± 0.0
Mix (− *Y. pestis*) *	1441.0	97.8 ± 1.5
Mix (+ *Y. pestis*) **	1392.0	94.5 ± 2.2

* Measurement of *Y. enterecolitica* in a mixture of three non-specific bacteria excluding *Y. pestis*. ** Measurement of *Y. enterecolitica* in a mixture of four non-specific bacteria including *Y. pestis*.

**Table 3:** Current response profile for *Y. enterocolitica* binding in human serum compared with other pathogenic bacteria, and the percentage of cross reactivity of non-specific bacteria (*n* = 3).

Bacteria Tested (10^7^ cfu mL^−1^)	Response (Current, nA)	GQD Immunosensor % Relative Activity
Yersinia enterocolitica	12.0	100.0 ± 2.0
Yersinia pestis	2.7	22.8 ± 2.0
Salmonella enteritidis	1.1	9.3 ± 0.3
Escherichia coli	1.0	8.3 ± 0.2
Bacillus anthracis	0.5	4.5 ± 0.1
Mix (− *Y. pestis*) *	10.5	87.5 ± 1.5
Mix (+ *Y. pestis*) **	9.1	75.8 ± 2.3

* Measurement of *Y. enterecolitica* in a mixture of three non-specific bacteria excluding *Y. pestis*. ** Measurement of *Y. enterecolitica* in a mixture of four non-specific bacteria including *Y. pestis*.
